# Wastewater dataset on the SARS-CoV-2 sublineages circulating in Central Arkansas, USA, post-COVID-19 pandemic

**DOI:** 10.1038/s41597-025-05100-x

**Published:** 2025-06-03

**Authors:** Volodymyr P. Tryndyak, Tetyana Kudlyk, Patricia Shores, Michelle M. Vanlandingham, Lisa Mullis, Luísa Camacho, Marli Azevedo, Camila S. Silva

**Affiliations:** 1 Division of Biochemical Toxicology, National Center for Toxicological Research, U.S. Food and Drug Administration, Jefferson, AR 72079-9501 USA; 2 Division of Microbiology, National Center for Toxicological Research, U.S. Food and Drug Administration, Jefferson, AR 72079-9501 USA

**Keywords:** SARS-CoV-2, Water resources

## Abstract

The ability of coronaviruses to adapt to new hosts and cause widespread disease outbreaks poses a significant threat to global public health systems and economies. The severity of the COVID-19 pandemic has emphasized the importance of studying coronaviruses and monitoring them in communities. We investigated SARS-CoV-2 and its genomic changes in wastewater influent sampled from two metropolitan areas in Arkansas, USA, between April 2020 and March 2024. The data presented here are a follow up report to our previous publication on the findings from the period of April 2020 to January 2022 and show the SARS-CoV-2 variants circulating between February 2022 and March 2024. The levels of viral RNA were measured by reverse-transcription quantitative polymerase chain reaction and targeted three SARS-CoV-2 genes (encoding ORF1ab polyprotein, ORF1ab; surface glycoprotein, S-protein; and nucleocapsid phosphoprotein, N-protein). The identity and genetic diversity of the virus were investigated using amplicon-based RNA sequencing. These data provide important information on SARS-CoV-2 evolution and help to understand the occurrence of COVID-19 outbreaks in the community.

## Background & Summary

Coronaviruses are enveloped RNA viruses widely distributed among mammals and birds. Their large genome (25–32 kilobases) exhibits high mutation rates and recombination frequencies, enabling them to adapt quickly to new hosts, change ecological niches and tissue tropism, induce the emergence of new strains, increase virulence, and expand their host range^1,2^. The close contact of humans with domestic animals, livestock, or wild animals provides the opportunity for interspecies transmission of coronaviruses and may potentially lead to further genetic changes for adapting to human hosts^1,2^.

The serious outcomes caused by severe acute respiratory coronavirus (SARS-CoV)^3^, Middle East respiratory coronavirus (MERS-CoV)^4^, and, more recently, SARS-CoV-2^5,6^ have increased awareness about coronaviruses as a potential threat to global public health and highlighted the importance of monitoring their evolution.

Wastewater surveillance has been implemented as an effective approach for the monitoring of SARS-CoV-2 and its genetic variants at the community level during the COVID-19 pandemic. The use of wastewater surveillance in pathogen detection has advanced considerably during the COVID-19 pandemic and its impact on the early warning of disease outbreaks has been emphasized^7–9^.

With the end of the global public health emergency for COVID-19 pandemic announced by the World Health Organization on 05 May 2023^10,11^, environmental surveillance efforts may play an important role on monitoring SARS-CoV-2 evolution, and potentially track the rise of new lineages, predict transmission dynamics, and prepare for potential future outbreaks.

We investigated SARS-CoV-2 and its genomic changes in wastewater influent sampled from two metropolitan areas in Arkansas, USA, throughout the major surges of COVID-19 cases. We previously reported the temporal dynamics of Alpha, Delta, and Omicron variants between April 2020 and January 2022^12^.

In the present study, the levels of SARS-CoV-2 RNA titers were estimated by quantification of three different regions of the virus’ genome (encoding ORF1ab, S-protein, and N-protein) in wastewater samples obtained from February 2022 through March 2024. The identity and genetic diversity of the virus were investigated using amplicon-based high-throughput sequencing. SARS-CoV-2 variants monitored in wastewater samples throughout the duration of the study closely reflected those found in COVID-19 patients from Arkansas during the same period (https://covid.cdc.gov/covid-data-tracker/#variant-proportions). Multiple Omicron variant sublineages were detected in wastewater samples collected in four study locations. The detection of the N-gene was consistently lower than that of ORF1ab- and S- genes during the period of outbreaks caused by Omicron sublineages in January 2022 – December 2023. Interestingly, the S-gene was not detected in January 2024, around the same time when Omicron JN.1 became predominant. Amplicon-based sequencing of viral RNA was shown to be an efficient method to track SARS-CoV-2 variants in wastewater over time and highlighted the association between the SARS-CoV-2 variants detected in wastewater and those found in clinical cases. The data in this article provide the scientific community insight on SARS-CoV-2 evolutionary dynamics and other aspects related to the occurrence of COVID-19 outbreaks.

## Methods

The methods used in the sample collection and processing, RNA extraction, and reverse-transcription quantitative polymerase chain reaction (RT-qPCR) are described in Silva *et al*.^12^.

**Fig. 1:**
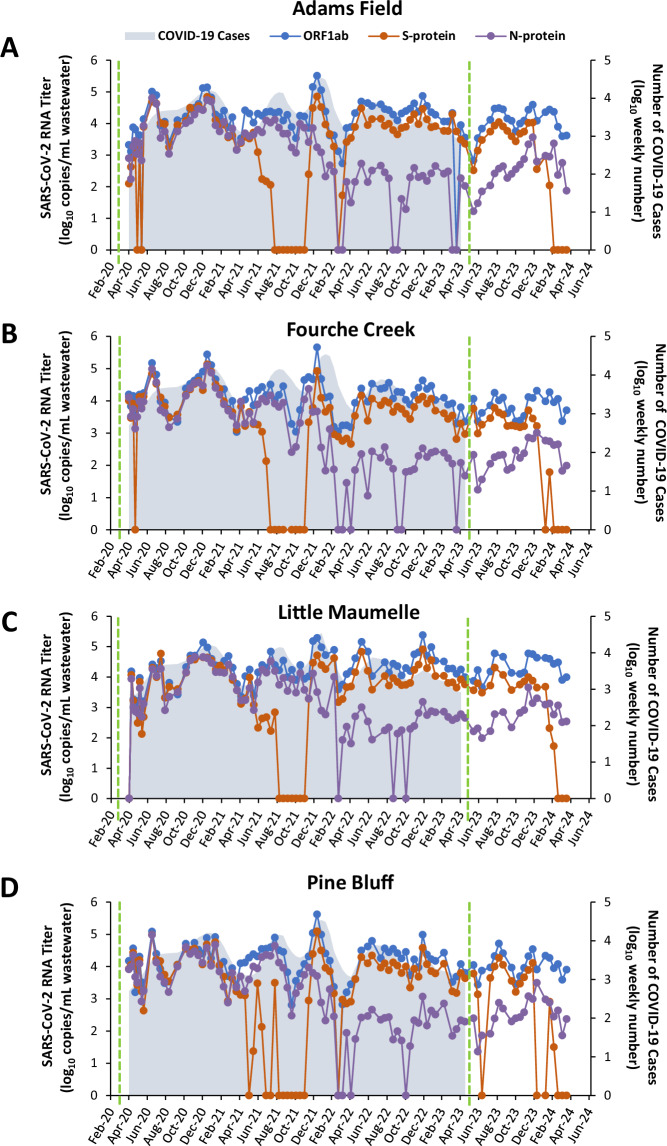
Temporal dynamics of SARS-CoV-2 genes in wastewater and COVID-19 case count in Little Rock, AR, USA and Pine Bluff, AR, USA. The left-hand y-axis presents the levels of the viral RNA by gene in wastewater samples in each treatment facility: Little Rock Water Reclamation Authority (A. Adams Field, B. Fourche Creek, C. Little Maumelle) and Pine Bluff Wastewater Utility (D. Pine Bluff) throughout the period of four years of wastewater surveillance (between April 2020 and April 2024). The data collected between April 2020 and January 2022 have been previously published^12^ and are plotted in this figure for informative purposes. The SARS-CoV-2 RNA titers were normalized to PMMoV and are presented as viral RNA log_10_ copy number/mL of wastewater shown per each gene (ORF1ab, S-protein, and N-protein). During the period of this study, wastewater samples were collected biweekly, with a few exceptions due to adverse conditions, such as inclement winter weather. Refer to Data File 2 in the Figshare repository for the full list of wastewater samples, respective collection dates and sites, and Ct values obtained through RT-qPCR. The right-hand y-axis shows the weekly number of new COVID-19 cases in the state of Arkansas during the same period (https://data.cdc.gov/Case-Surveillance/Weekly-United-States-COVID-19-Cases-and-Deaths-by-/pwn4-m3yp/about_data; accessed on 24 October 2024). The COVID-19 incidence data reflect positive RT-qPCR and/or rapid antigen results in individuals tested for SARS-CoV-2 and are shown as a 7-day moving average. Reporting of COVID-19 cases was discontinued on 11 May 2023, with the expiration of the COVID-19 public health emergency declaration. The green dashed vertical lines indicate the official start of the COVID-19 pandemic (11 March 2020) and the end of the COVID-19 public health emergency (05 May 2023), according to the World Health Organization.

The levels of SARS-CoV-2 RNA (Fig. 1) were measured by RT-qPCR in two hundred and one samples between 08 February 2022 and 16 April 2024. Pepper mild mottle virus (PMMoV) was also monitored to account for biases due not only to seasonal fluctuation, but also sampling, storage, and processing of wastewater.

The RNA from all 201 wastewater samples was sequenced, but not all yielded high-quality sequences due to challenges inherent to the nature of the samples, such as low viral abundance, the presence of RNA from a diverse range of other organisms, and inhibitory compounds that may interfere with RNA sequencing^13^. Sequencing data from 199 of the 201 samples were deposited in the National Center for Biotechnology Information (NCBI) public repository. Of these, 148 samples had sufficient coverage for phylogenetic analysis, and variant detection could be confidently completed for 177 samples.

### SARS-CoV-2 genome sequencing

An amplicon-based next-generation sequencing method was conducted on total RNA isolated from wastewater samples, as previously described^12^, with minor modifications in the preparation of the libraries. A DNase treatment step (using the TURBO DNA-free™ kit, Invitrogen, Waltham, MA, USA) was included in the KingFisher protocol for RNA extraction. RNA libraries were prepared using Illumina COVIDSeq Kits (Illumina, San Diego, CA, USA) coupled with SARS-CoV-2 ARTIC v.4 amplicon primer sets (Illumina), which contain 99 amplicons in two pools targeting the SARS-CoV-2 genome. Raw FASTQ sequencing files were uploaded to the Illumina BaseSpace^TM^ Sequence Hub (BSSH) cloud analysis system, trimmed to remove adapters using FASTQ Toolkit v.2.2.6 software, and submitted to the NCBI public repository. Trimmed FASTQ files were analyzed using the Illumina^®^ DRAGEN COVID Lineage App v4.0.6, which supports SARS-CoV-2 ARTIC V4 primer sets. Only samples with sufficient viral titer and meeting the following criteria were used in the variant calling and consensus sequence generation:

- *Percentage of amplicons with at least 90% coverage* (≥*1X*) *to enable variant calling and consensus sequence generation set to 50%;*
- *Variant Call (VC) Target Coverage was set to 50%;*
- *Filter out Mapping Quality (MAPQ)* <*20 from callable regions Browser Extensible Data (BED) option was enabled;*
- *Minimum variant depth (DP) coverage was set to 10;*
- *Minimum Variant Allele Frequency was set to 0.5;*
- * Remove human reads from FASTQ/Binary Alignment/Map (BAM) (de-hosting) using NCBI Sequence Read Archive (SRA) Human Read Scrubber option was enabled.*

The SARS-CoV-2 isolate Wuhan-Hu-1 sequence (NC_045512.2) was used as the reference. DRAGEN COVID Lineage output reports included alignment metrics, nucleotide and amino acid substitutions and deletions, consensus sequences in FASTA format, and variant call format (VCF) files.

**Fig. 2:**
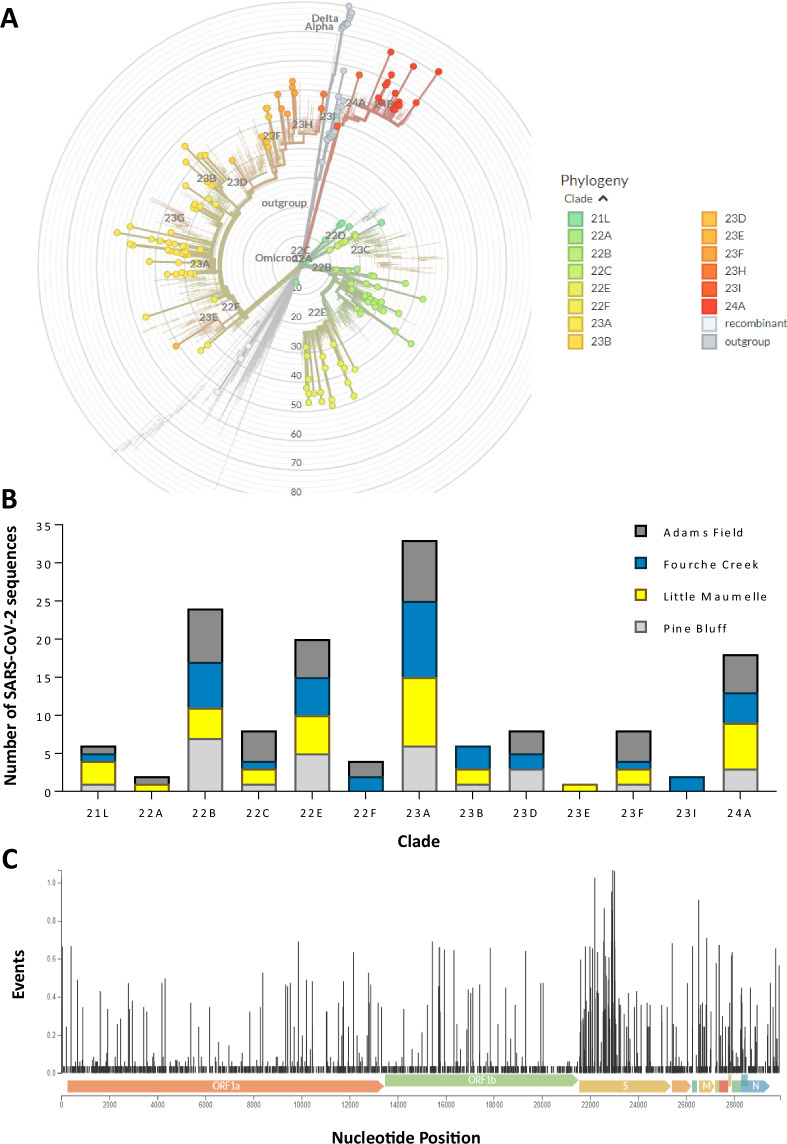
Diversity of SARS-CoV-2 viral genome consensus sequences obtained by RNA sequencing from wastewater samples collected at wastewater treatment facilities in Little Rock, AR, USA (Little Rock Water Reclamation Authority: Adams Field, Fourche Creek, and Little Maumelle) and Pine Bluff, AR, USA (Pine Bluff Wastewater Utility: Pine Bluff). (**A**) Phylogenetic tree showing the evolutionary relationship among SARS-CoV-2 lineages present in wastewater from these locations. Lines with dots corresponding to the legend color indicate samples sequenced in the study. Clades were assigned using the NextClade tool (https://clades.nextstrain.org)^14^. Refer to Data File 3 in the Figshare repository for more details on the samples and respective sequences used in the background of the phylogenetic tree. (**B**) Distribution of SARS-CoV-2 sequences within the phylogenetic clades by location between February 2022 and March 2024. (**C**) Rate of base pair substitutions within the nucleotides of SARS-CoV-2 genome. ‘Events’ refer to the count of genetic changes or mutations (e.g., substitutions, deletions, insertions) in the nucleotide or codon positions throughout the genome. These events are inferred using ancestral state reconstruction to identify where these changes occurred within the phylogenetic tree.

**Fig. 3:**
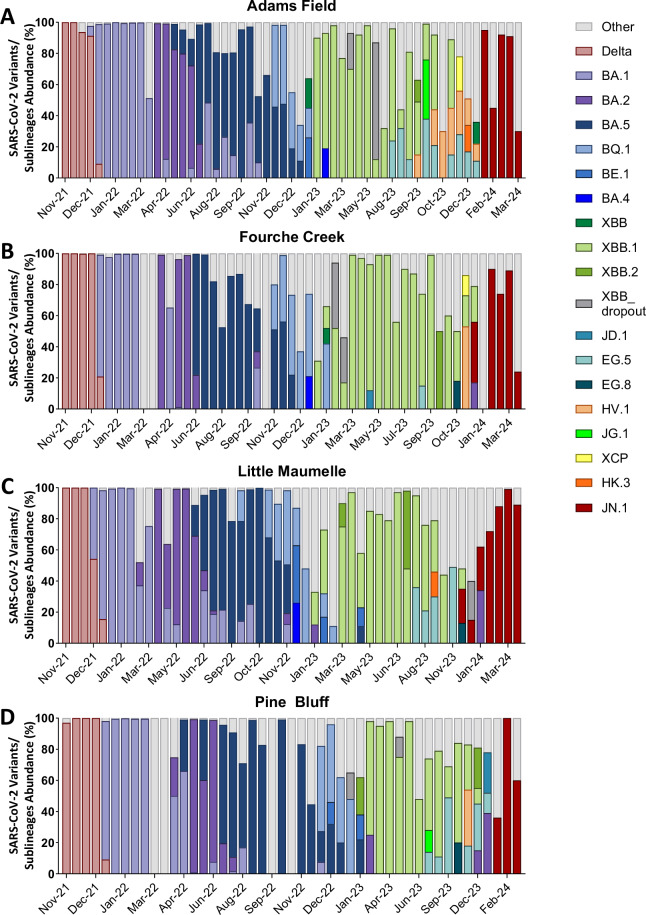
SARS-CoV-2 lineages based on amino acid changes in wastewater samples in Little Rock, AR, USA: Little Rock Water Reclamation Authority (A. Adams Field, B. Fourche Creek, C. Little Maumelle) and Pine Bluff, AR, USA: Pine Bluff Wastewater Utility (D. Pine Bluff) from November 2021 to March 2024. The y-axis displays the relative abundance of SARS-CoV-2 lineages estimated in wastewater samples by using the bioinformatic package Freyja (https://github.com/andersen-lab/Freyja)^13^. “Other” refers to SARS-CoV-2 genomic sequences that cannot be assigned to any of the known Pango lineages or that constitute less than 5% abundance of the circulating lineages. Data File 4 in Figshare repository shows the relative abundance of SARS-CoV-2 lineages based on amino acid changes in these wastewater samples. The data between November 2021 and January 2022 have been previously published^12^ and are included in this figure to illustrate the transition from SARS-CoV-2 Delta to Omicron variants.

Next, SARS-CoV-2 consensus sequences with >50% genome coverage and ≥10X per base coverage were uploaded and analyzed by NextClade tool v3.9.1 at https://clades.nextstrain.org/^14^ (Fig. 2 and Data File 3). Trimmed FASTQ files were uploaded to the bioinformatics package Freyja (https://github.com/andersen-lab/Freyja, Andersen Lab at the Scripps Research Institute, La Jolla, CA)^13^, and default parameters (https://andersen-lab.github.io/Freyja/src/usage/demix.html) were applied to estimate the relative abundance of SARS-CoV-2 lineages in the wastewater samples (Fig. 3 and Data File 4).

## Data Records

The data generated by RNA sequencing are available at the NCBI public repository via NCBI SRA: SRP389725^15^ under the BioProject accession number PRJNA865728, which is a component of the GenomeTrakr^16^ umbrella for SARS-CoV-2 wastewater data. The datasets include trimmed FASTQ sequencing data and RT-qPCR cycle threshold (Ct) values for Orf1ab and PMMoV.

Additional information was shared in the Figshare repository (10.6084/m9.figshare.28071389)^17^, including NCBI SRA accession numbers and metadata (Data File 1), mean Ct values of endogenous control (PMMoV) and SARS-CoV-2 genes (Orf1ab, S-protein, and N-protein) detected by RT-qPCR of the wastewater samples between February 2022 and April 2024 (Data File 2), and data on SARS-CoV-2 lineages/clades (Data File 3) and their relative abundance (Data File 4) detected through RNA sequencing of the wastewater samples between February 2022 and March 2024. The lineage data were generated using Illumina DRAGEN COVID Lineage software, and the most abundant variants and consensus sequences detected in each sample are shown (Data File 3). This software aligns SARS-CoV-2 sequence reads to a reference genome to generate a consensus genome sequence and identify mutations, including amino acid substitutions and deletions. It then performs lineage/clade classification using Pangolin and/or NextClade. VCF files from this study were deposited into the European Variation Archive (EVA) database^18^ at EMBL-EBI under project accession number PRJEB87405^19^ for open public access.

Data File 4 shows the relative abundance of SARS-CoV-2 lineages based on amino acid changes in wastewater samples collected at the wastewater treatment facilities in Little Rock, AR, USA (Little Rock Water Reclamation Authority: Adams Field, Fourche Creek, and Little Maumelle) and Pine Bluff, AR, USA (Pine Bluff Wastewater Utility: Pine Bluff) from November 2021 to March 2024. The data were obtained by using Freyja (https://github.com/andersen-lab/Freyja)^13^.

## Technical Validation

PMMoV was used as internal reference and human fecal control^12,20,21^ because it is stable, abundant in domestic wastewater, and well-established as an enteric virus process indicator^22,23^. In addition, a known amount of an exogenous RNA process control (TaqMan® Universal RNA Spike In/Reverse Transcription (Xeno) Control, Applied Biosystems, Foster City, CA, USA) was added to each sample and carried through the workflow to monitor RNA recovery and RT-qPCR inhibition. An RNA extraction blank was also included to monitor for contamination^12^.

Serial dilutions of viral RNA extracted from Milli-Q water seeded with a known quantity of the heat-inactivated SARS-CoV-2 isolate USA-WA1/2020 (NR-52286, Biodefense and Emerging Infections Research Resources Repository, Manassas, VA, USA)^24^ were used to generate standard curves for each TaqMan 2019-nCoV assay^12^.

## Data Availability

No custom code was used in the present study for the curation and/or validation of the datasets. The software programs used in the present study are publicly available^13,14^. DRAGEN COVID Lineage is available on the Illumina’s BaseSpace™ Sequence Hub (https://basespace.illumina.com/). The NextClade tool v3.9.1 at https://clades.nextstrain.org/14 is publicly hosted on GitHub (https://github.com/nextstrain/nextclade), and all source code is freely available under the terms of MIT open-source license. The bioinformatic package Freyja^13^ is publicly available at https://github.com/andersen-lab/Freyja, under BSD-2-Clause license.
